# Use of Metabolic Scores and Lipid Ratios to Predict Metabolic Dysfunction-Associated Steatotic Liver Disease Onset in Patients with Inflammatory Bowel Diseases

**DOI:** 10.3390/jcm14092973

**Published:** 2025-04-25

**Authors:** Ludovico Abenavoli, Giuseppe Guido Maria Scarlata, Massimo Borelli, Evelina Suraci, Raffaella Marasco, Maria Imeneo, Rocco Spagnuolo, Francesco Luzza

**Affiliations:** 1 Department of Health Sciences, University “Magna Graecia”, 88100 Catanzaro, Italy; giuseppeguidomaria.scarlata@unicz.it (G.G.M.S.); spagnuolo@unicz.it (R.S.); luzza@unicz.it (F.L.); 2 UMG School of PhD Programmes “Life Sciences and Technologies”, University “Magna Graecia”, 88100 Catanzaro, Italy; massimo.borelli@unicz.it; 3 Inflammatory Bowel Disease Unit, Renato Dulbecco University Hospital, 88100 Catanzaro, Italy; e.suraci@libero.it (E.S.); raffaellamarasco1@gmail.com (R.M.); graziaimeneo@hotmail.it (M.I.)

**Keywords:** biomarkers, accuracy, diagnosis, Crohn’s disease, ulcerative colitis, fatty liver disease

## Abstract

**Background/Objectives:** Metabolic dysfunction-associated steatotic liver disease (MASLD) is increasingly recognized in inflammatory bowel disease (IBD) patients due to chronic inflammation and metabolic disturbances. However, reliable non-invasive biomarkers for MASLD prediction in this population are lacking. This study evaluated the predictive value of metabolic scores and lipid ratios for MASLD onset in IBD patients. **Methods:** An observational retrospective study was conducted on 358 IBD patients at the “Renato Dulbecco” Teaching Hospital in Catanzaro, Italy, in a period between 1 January 2021 and 31 December 2024. Clinical and laboratory data, including metabolic scores and lipid ratios, were analyzed using the chi-square and Kruskal–Wallis tests as appropriate. Post hoc comparisons were conducted using Dunn’s test. Receiver operating characteristic analysis assessed their predictive accuracy for MASLD. *p* < 0.05 was considered significant. **Results:** IBD-MASLD patients had a significantly higher body mass index (BMI, 27 ± 4 vs. 22 ± 2 kg/m^2^; *p* < 0.001), waist circumference (100 ± 11 vs. 85 ± 4 cm; *p* < 0.001), other anthropometric parameters, metabolic scores, and lipid ratios than IBD-only patients. The metabolic score for insulin resistance [METS-IR, area under curve (AUC = 0.754)] and waist circumference (AUC = 0.754) exhibited the highest predictive accuracy, followed by the lipid accumulation product (LAP, AUC = 0.737), BMI (AUC = 0.709), and triglyceride/high-density lipoprotein (TG/HDL, AUC = 0.701). Insulin resistance scores, including the homeostasis model assessment of insulin resistance (AUC = 0.680) and triglyceride-glucose index (AUC = 0.674), were of moderate predictive use. The visceral adiposity index (AUC = 0.664) and low-density lipoprotein/high-density lipoprotein (AUC = 0.656) showed lower discriminative ability, while the fibrosis-4 index (AUC = 0.562) had the weakest diagnostic performance. **Conclusions:** Our findings suggest that MASLD in IBD is primarily driven by cardiometabolic dysfunction. The introduction of the METS-IR, LAP, and TG/HDL into clinical assessments of IBD patients could prove useful in preventing liver and cardiovascular complications in this setting.

## 1. Introduction

### 1.1. Interplay Between IBD and MASLD

Metabolic dysfunction-associated steatotic liver disease (MASLD) is the updated terminology that has replaced non-alcoholic fatty liver disease (NAFLD), and was also previously referred to as metabolic dysfunction-associated fatty liver disease (MAFLD). This redefinition reflects a shift toward emphasizing the underlying metabolic dysfunction. MASLD has emerged as a major global health concern due to its rising prevalence and strong association with metabolic syndrome and cardiovascular disease [1]. This condition is estimated to affect approximately 30% of the global population, with a higher burden in individuals with obesity, insulin resistance, and dyslipidemia [2]. Without proper management, MASLD may advance to more severe liver conditions, such as metabolic dysfunction-associated steatohepatitis, progressive fibrosis, cirrhosis, and eventually hepatocellular carcinoma. This progression poses a growing global health concern due to its increasing prevalence and potential for serious complications [3]. Despite a growing understanding of MASLD pathophysiology, its early detection remains suboptimal, highlighting the urgent need to establish reliable methods for early detection, including non-invasive biomarkers [4]. Inflammatory bowel diseases (IBDs), which include Crohn’s disease (CD) and ulcerative colitis (UC), are chronic immune-mediated disorders characterized by relapsing-remitting inflammation of the gastrointestinal tract [5]. Worldwide, the incidence and prevalence of IBD have shown a consistent upward trend—from 4.22 per 100,000 in 1990 to 4.45 per 100,000 in 2021, especially in newly industrialized nations, placing considerable strain on healthcare systems and negatively impacting patients’ quality of life [6]. IBD is associated with systemic inflammation, gut dysbiosis, and metabolic disturbances, which may predispose affected individuals to extraintestinal manifestations, including liver diseases such as MASLD [7]. Emerging evidence suggests a pathogenetic link between IBD and MASLD, with IBD patients having an increased risk of developing MASLD. Chronic inflammation, intestinal dysbiosis, increased gut permeability, and altered lipid metabolism are key contributors to hepatic steatosis in this population [8,9]. Despite these associations, the precise mechanisms linking IBD and MASLD remain incompletely understood, and reliable, non-invasive methods for predicting MASLD onset in IBD patients are lacking [10].

### 1.2. Diagnostic Tools

Given the growing burden of MASLD in the IBD population and the heterogeneity in the application of current diagnostic tools, it is necessary to identify new non-invasive biomarkers for the prediction of MASLD onset in these patients. Traditional liver function tests lack sensitivity and specificity for early-stage MASLD [11]. For instance, hepatic ultrasound is an accurate and reliable technique for detecting only moderate and severe fatty liver. Moreover, it is characterized by both intra- and inter-observer variability [12]. Another option would be transient elastography, a validated, non-invasive method for assessing hepatic steatosis. However, it does have its limitations, including its difficulties in detecting acute liver failure, hepatic congestion, cholestasis, and obesity, and its reliance on operator experience [13]. In the last decade, several non-invasive scoring systems—such as the fibrosis-4 (FIB-4) index, NAFLD fibrosis score (NFS), and enhanced liver fibrosis test—have been developed to assess liver fibrosis, a key prognostic factor in MASLD. However, these tools were primarily validated in the general population and may require adjustment for application in patients with IBD, considering the specific inflammatory profile [9,14]. In addition, gut-derived biomarkers, including microbial metabolites and biomarkers of gut permeability (e.g., zonulin), may be included in the pathogenic mechanism between IBD-related dysbiosis and MASLD development. Further studies are warranted in order to validate the application of these cross-disease biomarkers and to develop integrated diagnostic algorithms that are tailored for patients affected by both IBD and MASLD [15].

### 1.3. Aim of the Study

For this reason, the present study aimed to investigate the use of metabolic scores and lipid ratios to predict MASLD onset in patients with IBD.

## 2. Materials and Methods

### 2.1. Study Design

An observational retrospective study was conducted involving 358 patients diagnosed with IBD according to established international guidelines [16] at the “Renato Dulbecco” Teaching Hospital in Catanzaro, Italy. The study period extended from 1 January 2021, to 31 December 2024. The inclusion criteria required participants to be 18 years of age or older and to have received liver imaging at the time of hospital admission, while the exclusion criteria comprised the following: (i) liver cirrhosis, (ii) any form of cancer, and (iii) pregnancy or breastfeeding. All patients underwent liver evaluation via abdominal ultrasound, following the protocols established in our previous studies [17,18,19,20]. Likewise, fatty liver disease evaluation was performed according to international guidelines. In summary, in order to diagnose NAFLD, we evidenced hepatic steatosis via abdominal ultrasound, accompanied by the exclusion of significant alcohol consumption (generally defined as less than 210 g per week for men and 140 g per week for women). In addition, other secondary causes of hepatic fat accumulation were ruled out, including chronic viral hepatitis, drug-induced liver injury, and autoimmune chronic liver disease. Regarding MASLD diagnosis, we detected hepatic steatosis via ultrasound in combination with at least one of the following five cardiometabolic risk indicators: (i) a body mass index (BMI) equal to or exceeding 25 kg/m^2^ or an increased waist circumference (higher than 94 cm in men and 80 cm in women); (ii) elevated fasting glucose levels (≥100 mg/dL) or a confirmed diagnosis of type 2 diabetes mellitus (T2DM), including those receiving antidiabetic therapy; (iii) elevated blood pressure (≥135/85 mmHg) or ongoing treatment with antihypertensive medications; (iv) raised triglyceride (TG) levels (≥150 mg/dL) or current use of lipid-lowering agents; and (v) reduced high-density lipoprotein (HDL) cholesterol (≤40 mg/dL in men and ≤50 mg/dL in women) or pharmacological treatment targeting dyslipidemia [21,22].

### 2.2. Demographic, Anthropometric, and Clinical Data Collection

Demographic, anthropometric, and clinical data were extracted through a thorough review of the patients’ medical records, as follows: age, gender, waist circumference, smoking habit, age at onset, disease duration, type of IBD, Harvey–Bradshaw Index [23], Full Mayo Score [24], IBD extension and phenotype [25], presence of extraintestinal manifestations, presence and grade of liver steatosis [26], presence of cardiometabolic comorbidities such as T2DM, overweight or obesity, hypertension, dyslipidemia, and history of surgery.

### 2.3. Laboratory and Treatment Data Collection

Furthermore, we evaluated the following laboratory parameters: platelet count, alanine aminotransferase (ALT), aspartate aminotransferase (AST), total cholesterol, low-density lipoprotein (LDL), HDL, TG, fasting blood glucose, fasting insulinemia, fecal calprotectin, and C-reactive protein (CRP). We also collected data about treatment: use of salicylates, azathioprine, >3 cycles of steroids, use of biological therapy with anti-tumor-necrosis factor alpha (TNF-α), vedolizumab, ustekinumab, >1 biological drugs, duration of current biological therapy, and duration of total biological therapy.

### 2.4. Anthropometric, Clinical Scores, and Ratios Calculation

Finally, we calculated some anthropometric and clinical scores and ratios, such as the BMI [27], lipid accumulation product (LAP) [28], visceral adiposity index (VAI) [29], homeostasis model assessment of insulin resistance (HOMA-IR) [30], metabolic score for insulin resistance (METS-IR) [31], TG/HDL [32], triglyceride-glucose index (TyG) [33], LDL/HDL [34], and FIB-4 index [35].

### 2.5. Statistical Analysis

Continuous variables are expressed as mean ± standard deviation (SD), while categorical variables are reported as absolute frequencies and percentages. Statistical comparisons of the categorical and continuous data were performed using the chi-square (χ^2^) and Kruskal–Wallis tests, respectively, assuming non-normal distribution verified by the Shapiro–Wilk test. Post hoc comparisons were conducted using Dunn’s test. To assess whether the proportion of females differed significantly between groups, two-sample tests for equality of proportions with continuity correction were performed, excluding the patients in the IBD-NAFLD group due to the limited sample size and lack of female gender representation. The predictive performance of metabolic scores for MASLD onset in IBD patients was evaluated through receiver operating characteristic (ROC) curve analysis using the pROC package [36]. A *p*-value < 0.05 was considered indicative of statistical significance. All analyses were performed with R software version 4.1.2 (R Foundation for Statistical Computing) and JASP software version 0.19.3.0 (JASP Team) [37].

## 3. Results

Table 1 shows the clinical features of the enrolled patients, divided into different groups. The patients with IBD-MASLD were significantly older than those with IBD-NAFLD and the IBD cohort (52 ± 12 vs. 37 ± 15 vs. 46 ± 16 years; *p* < 0.001). At the same time, the male gender was more prevalent in the IBD-NAFLD group than in the other two groups [*n* = 13 (93%) vs. *n* = 48 (72%) vs. *n* = 151 (54%), *p* < 0.001]. The IBD-MASLD patients showed significantly higher values of BMI (27 ± 4 vs. 22 ± 2 vs. 24 ± 4 kg/m^2^, *p* < 0.001), waist circumference (100 ± 11 vs. 85 ± 4 vs. 90 ± 11 cm, *p* < 0.001), LAP (50 ± 29 vs. 18 ± 5 vs. 31 ± 22; *p* < 0.001), and VAI (2 ± 1 vs. 1 ± 0.2 vs. 1 ± 1; *p* < 0.001) than the IBD-NAFLD and IBD groups, respectively. Regarding the disease characteristics, the disease duration did not differ significantly among the groups (*p* = 0.369), but age at onset was higher in the IBD-MASLD patients compared to the IBD-NAFLD and IBD groups (38 ± 15 vs. 22 ± 10 vs. 34 ± 15 years, *p* < 0.001), respectively. The distribution of CD and UC was similar among the groups [*n* = 6 (43) vs. *n* = 22 (33) vs. *n* = 97 (35); *p* = 0.772], with a higher prevalence of ileal location for CD [*n* = 4 (67) vs. 10 (45) vs. 57 (59); *p* = 0.736] and pancolitis for UC [*n* = 4 (50) vs. *n* = 21 (47) vs. *n* = 85 (47); *p* = 0.075], respectively. Similarly, the disease activity indices, including the Harvey–Bradshaw Index for CD and the Mayo Score for UC, did not show significant differences between the groups. The IBD-MASLD group showed a significantly higher degree of severity for liver steatosis [*n* = 7 (10% vs. *n* = 1 (7%); *p* < 0.001] than the IBD-NAFLD patients. Furthermore, the patients with IBD-MASLD had a significantly higher prevalence of T2DM [*n* = 9 (13%) vs. 9 (3%) vs. 0; *p* = 0.002] and hypertension [*n* = 24 (36) vs. *n* = 36 (13) vs. 0; *p* < 0.001] compared to the IBD-NAFLD and IBD groups, respectively. Dyslipidemia was observed in *n* = 10 (15%) of IBD-MASLD patients but was not significantly different from the other groups (*p* = 0.11).

As for the laboratory findings and scores, both the ALT and AST levels were significantly higher in the IBD-MASLD group compared to the IBD-NAFLD and IBD groups (27 ± 26 vs. 24 ± 9 vs. 19 ± 11 UI/L, *p* = 0.001 and 23 ± 12 vs. 25 ± 9 vs. 20 ± 13 UI/L, *p* = 0.017; respectively). TG levels were significantly elevated in the IBD-MASLD patients (118 ± 52 vs. 82 ± 15 vs. 95 ± 46 mg/dL; *p* < 0.001), and HDL cholesterol was lower (49 ± 13 vs. 58 ± 12 vs. 57 ± 15 mg/dL; *p* = 0.021). Insulin resistance indices, including the HOMA-IR (3 ± 2 vs. 1.5 ± 0.4 vs. 2 ± 1.5, *p* < 0.001), METS-IR (40 ± 8 vs. 31 ± 4 vs. 33 ± 6; *p* < 0.001), and TyG index (8 ± 0.4 vs. 8 ± 0.2 vs. 8 ± 0.5; *p* < 0.001), were significantly higher in the IBD-MASLD patients. Likewise, TG/HDL (3 ± 2 vs. 1.5 ± 0.5 vs. 2 ± 1; *p* < 0.001) and LDL/HDL (3 ± 1 vs. 2 ± 1 vs. 2 ± 1; *p* < 0.001) were significantly higher in the subjects with both IBD and MASLD, while the FIB-4 was not significantly different among the groups (1 ± 0.5 vs. 1 ± 0.5 vs. 1 ± 1; *p* = 0.546). Finally, as for the use of medications, there were no significant differences in the use of salicylates, azathioprine, steroids, or biological therapies among the groups, as reported in Table 2.

Table 3 shows the results of the Dunn’s post hoc analysis to compare the variables among the groups. Specifically, significant differences were observed in age and BMI between the IBD-NAFLD and IBD-MASLD groups. The IBD-NAFLD group was significantly younger (37 ± 15 years) compared to the IBD-MASLD group (52 ± 12 years, *p* < 0.001) and the IBD population (46 ± 16 years, *p* < 0.001). Regarding BMI, the patients in the IBD-MASLD group had a significantly higher BMI (27 ± 4 kg/m^2^) compared to both the IBD-NAFLD group (22 ± 2 kg/m^2^, *p* < 0.001) and the IBD group (24 ± 4 kg/m^2^, *p* < 0.001). The IBD-MASLD patients also had a significantly larger waist circumference (100 ± 11 cm) compared to the IBD-NAFLD (85 ± 4 cm, *p* < 0.001) and IBD (90 ± 11 cm, *p* < 0.001) groups. At the same time, LAP was significantly higher in the IBD-MASLD group (50 ± 29) compared to the IBD-NAFLD group (18 ± 5, *p* < 0.001) and also higher than the IBD group (31 ± 22, *p* < 0.001), while the VAI was significantly higher in the IBD-MASLD group (2 ± 1) than in the IBD-NAFLD group (0.9 ± 0.2, *p* = 0.005) and the IBD group (1 ± 1, *p* < 0.001). Regarding the disease characteristics, the age at onset of IBD was significantly younger in the IBD-NAFLD group (22 ± 10 years) compared to the IBD-MASLD group (38 ± 15 years, *p* < 0.001) and the IBD group (34 ± 15 years, *p* = 0.025). Disease activity, as assessed by the Harvey–Bradshaw index for CD, showed a significant difference between IBD-NAFLD and IBD groups (3 ± 2 vs. 6 ± 3; *p* = 0.041), while the Mayo score for UC, showed no significant differences between the groups. The laboratory findings revealed that both the ALT and AST levels were significantly higher only in the IBD-MASLD vs. IBD groups comparison (27 ± 26 vs. 19 ± 11 UI/L; *p* < 0.001 and 23 ± 12 vs. 20 ± 13 UI/L; *p* = 0.014, respectively), while TG levels were significantly higher in the IBD-MASLD group (118 ± 52 mg/dL) compared to the IBD-NAFLD (82 ± 15 mg/dL, *p* = 0.048) and IBD alone (95 ± 46 mg/dL, *p* < 0.001) groups. The IBD-MASLD group also had significantly lower HDL levels (49 ± 13 mg/dL) compared to the IBD group (57 ± 15 mg/dL, *p* = 0.006), but fasting insulinemia levels were significantly elevated in the IBD-MASLD group (11 ± 8 mg/dL) compared to the IBD group (8 ± 5 mg/dL, *p* < 0.001). At the same time, fasting blood glucose levels were significantly higher in the IBD-MASLD population than in the IBD-only (93 ± 21 vs. 86 ± 14 mg/dL; *p* < 0.001). Regarding the different scores applied, the HOMA-IR was significantly higher in the IBD-MASLD group (3 ± 2) compared to the IBD group (2 ± 1.5, *p* < 0.001), and the METS-IR was significantly higher in the IBD-MASLD group (40 ± 8) compared to the IBD group (33 ± 6, *p* < 0.001) and to the IBD-NAFLD group (31 ± 4, *p* < 0.001). The TG/HDL ratio was significantly higher in the IBD-MASLD group (3 ± 2) than in the IBD-NAFLD group (1.5 ± 0.5, *p* = 0.028) and IBD (2 ± 1, *p* < 0.001). Moreover, the TyG was significantly higher in the IBD-MASLD patients than in the IBD patients (8 ± 0.4 vs. 8 ± 0.5; *p* < 0.001). At the same time, the LDL/HDL ratio was also higher in the IBD-MASLD group (3 ± 1) compared to the IBD group (2 ± 1, *p* < 0.001), though not significantly different from the IBD-NAFLD group (2 ± 1; *p* = 0.265). Finally, there were no significant differences in the duration of biological therapy among the groups.

**Figure 1:**
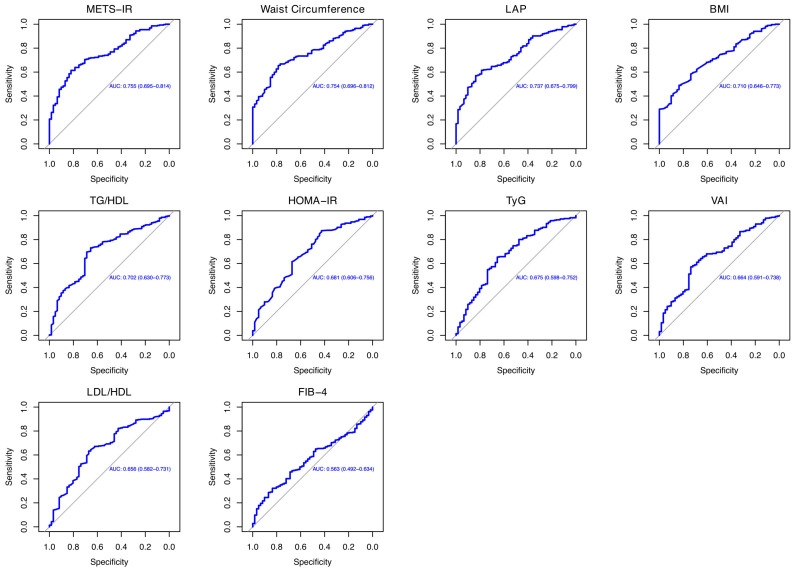
Representation of ROC analysis.

The comparison of the proportion of females in the IBD-MASLD group versus the overall sample showed no statistically significant difference (40.8% vs. 27.9%, χ^2^ = 3.13, *df* = 1, *p* = 0.07). Similarly, the proportion of females in the IBD group compared to the overall cohort was not significantly different (40.8% vs. 45.1%, χ^2^ = 1.04, *df* = 1, *p* = 0.306). These findings indicate that the gender distribution did not differ significantly across the analyzed groups. Finally, a ROC analysis was performed to assess whether metabolic and cholesterol ratios can predict MASLD onset in IBD patients, as shown in Table 4 and Figure 1. Among the evaluated indices, METS-IR and waist circumference exhibited the highest diagnostic accuracy, both achieving an AUC = 0.754 (95% confidence interval [CI]: 0.694–0.814 and 0.696–0.811, respectively). Specifically, METS-IR demonstrated a sensitivity of 70.5% and a specificity of 70.4% at a cut-off value of 36.52, while waist circumference showed a lower sensitivity (66.7%) but a higher specificity (77%) at a cut-off of 93.55 cm. The LAP followed closely with an AUC = 0.737 (95% CI: 0.675–0.798), a sensitivity of 61.5%, and a specificity of 78.6% at a cut-off of 29.8. Similarly, BMI exhibited moderate diagnostic accuracy with an AUC = 0.709, a sensitivity of 62.2% (95% CI: 0.646–0.773), and a specificity of 68.8% at a threshold of 24.99 kg/m^2^. Likewise, TG/HDL showed an AUC = 0.701 (95% CI: 0.630–0.773) with a sensitivity of 69.5% and a specificity of 68.8% at a cut-off of 1.91. Biomarkers of insulin resistance, including the HOMA-IR and TyG, displayed lower AUC values of 0.680 (95% CI: 0.605–0.755) and 0.674 (95% CI: 0.598–0.751), respectively. The HOMA-IR had a sensitivity of 61.5% and a specificity of 67.2% at a cut-off of 1.65, whereas the TyG index showed a sensitivity of 65.3% and a specificity of 65.5% at a cut-off of 8.33. Other indices, including the VAI and LDL/HDL, demonstrated relatively lower discriminative ability, with AUC values of 0.664 (95% CI: 0.590–0.737) and 0.656 (95% CI 0.581–0.730), respectively. Their sensitivity and specificity were similar and ranged from 62.8–63.2% to 67.2%, with cut-off values of 1.22 for the VAI and 1.96 for LDL/HDL, respectively. Finally, the FIB-4 exhibited the lowest diagnostic accuracy, with an AUC = 0.562 (95% CI 0.491–0.634), a sensitivity of 46.8%, and a specificity of 67.2% at a cut-off of 10.8.

## 4. Discussion

### 4.1. Comparison Between the Different Cohorts

Our study offers an analysis of anthropometric, clinical, and cardiometabolic profiles among IBD patients, highlighting key differences between those with MASLD, those with IBD-related NAFLD, and individuals with IBD without hepatic involvement. By analyzing key metabolic features, liver function parameters, and disease severity, it offers valuable insights into the distinct pathophysiological features of these patient groups. Our findings highlight significant differences in demographic, anthropometric, metabolic, and hepatic features, emphasizing the complex interplay between IBD and fatty liver disease. Patients with IBD-MASLD were significantly older with later disease onset than those in the IBD-NAFLD and IBD cohorts, suggesting a potential age-related metabolic burden that predisposes these individuals to MASLD [38]. Moreover, male gender was most frequent in the IBD-NAFLD group. Indeed, fatty liver disease and its related cardiometabolic comorbidities exhibit gender-specific disparities, showing a higher prevalence in males beyond their fifth decade of life [39]. Obesity-related anthropometric parameters, including BMI, waist circumference, LAP, and VAI, were significantly elevated in the IBD-MASLD patients, reinforcing the central role of overweight/obesity in MASLD pathogenesis [1]. Indeed, excess fat tissue leads to insulin resistance, causing fat to accumulate in the liver and disrupting normal lipid metabolism. At the same time, the low-grade inflammation driven by proinflammatory cytokines like TNF-α and interleukin-6 worsens liver damage and fibrosis. Additionally, imbalances in adipokines, such as decreased adiponectin and elevated leptin, further disrupt metabolism and accelerate liver disease progression [40]. The distribution of CD and UC was comparable among the groups, suggesting that MASLD development is not necessarily ascribed to the type of IBD but rather that it depends on associated metabolic risk factors [18,41]. Liver steatosis severity was significantly higher in the IBD-MASLD group compared to the IBD-only group, which is in accordance with the recent literature that underlines significant differences between patients with fatty liver disease and control subjects [42]. Moreover, the significantly higher prevalence of cardiometabolic comorbidities, including T2DM and hypertension, in the IBD-MASLD patients supports the hypothesis that metabolic syndrome plays a pivotal role in MASLD development within the IBD population [3,19]. The observed significant differences in ALT levels and lipid profiles among the IBD-MASLD, IBD-NAFLD, and IBD groups align with findings from our previous investigation [15]. Specifically, elevated ALT levels in the IBD-MASLD group suggest a higher degree of hepatic injury, because metabolic dysfunction exacerbates liver inflammation [43]. Furthermore, the significantly higher TG levels and lower HDL cholesterol in the IBD-MASLD patients reinforce the well-established link between metabolic syndrome and MASLD. These findings are in line with prior studies demonstrating that IBD patients with metabolic dysfunction exhibit a more atherogenic lipid profile, increasing their risk of cardiovascular complications [44]. Moreover, the significant reduction in HDL levels aligns with evidence suggesting that chronic inflammation and insulin resistance contribute to change lipid metabolism in both IBD and MASLD [45]. Overall, this finding supports the central role of the use of MASLD nomenclature in clinical practice in order to better identify individuals with fatty liver disease and associated cardiometabolic conditions, thus ensuring that they receive multidisciplinary management [22]. At the same time, it is important to consider that the cause–effect relationship between the pharmacological treatment of IBD and fatty liver disease remains debated. On the one hand, corticosteroids and immunosuppressants undergo significant hepatic metabolism, which may contribute to the development of MASLD [20]. On the other hand, the role of biologic therapies in these patients is not fully understood, with some evidence suggesting that anti-TNF-α agents may exert a protective effect against hepatic fat accumulation [17].

### 4.2. Metabolic Scores and Lipid Ratios Accuracy Analysis

In evaluating the predictive capacity of various metabolic scores and lipid ratios for MASLD onset in patients with IBD, our analysis confirmed the accuracy of diagnostic indicators already included in the MASLD diagnostic criteria, including waist circumference and BMI [22]. In fact, a recent cross-sectional study of a large cohort of patients showed that these two variables were associated with an increased risk of MASLD in the general population. Specifically, waist circumference showed strong discriminatory activity in highlighting the risk of MASLD, with an AUC = 0.802 [46]. Moreover, an increase in these two variables is associated with increased mortality in patients with MASLD [47]. However, while BMI is widely used to assess general obesity, it does not differentiate between fat and lean mass, which may limit its effectiveness in predicting metabolic disorders compared to measures like waist circumference [48]. For the latter, its inclusion in clinical assessments is crucial, as it serves as a simple yet effective indicator of central obesity and related metabolic risks [49]. Others anthropometric scores, such as the LAP and VAI showed an AUC = 0.737 and =0.664 when predicting MASLD onset. Regarding the first variable, the LAP showed an AUC = 0.698 in predicting NAFLD onset in a small cohort of obese children and an AUC = 0.843 in Chinese young individuals of both genders [50,51]. As for the use of the VAI, a recent meta-analysis based on nine studies showed an AUC = 0.790 for the assessment of MAFLD risk [52]. Additionally, a modified VAI was applied in a population with different levels of BMI. Specifically, the modified VAI and BMI were independently associated with the risk of MASLD, showing a better diagnostic ability to predict MASLD risk in the female gender [53]. Insulin resistance scores, such as the METS-IR, HOMA-IR, and TyG index, showed an accuracy between 0.674 and 0.754, with the METS-IR showing the highest accuracy to predict MASLD onset in our cohort of IBD patients. When comparing these findings to the existing literature, it is noteworthy that the METS-IR has been used as a reliable biomarker for insulin sensitivity and has shown strong correlations with NAFLD in patients with T2DM, showing an accuracy of 0.781 [54]. Additionally, in a study assessing the predictive utility of various insulin resistance indices for all-cause and cardiovascular mortality, the METS-IR demonstrated significant predictive value, further supporting its clinical relevance [55]. Furthermore, according to Lee et al., the METS-IR demonstrates higher accuracy than the HOMA-IR in predicting the onset of NAFLD and performs at least as well as the HOMA-IR in identifying existing cases [56]. The HOMA-IR was considered in the MAFLD diagnostic criteria, but not in the diagnostic algorithm of MASLD [53]. However, our study showed intermediate accuracy in MASLD prediction (AUC = 0.680), lower than other analyses performed in the recent literature that showed an AUC = 0.792 and a positive association between MASLD and higher HOMA-IR levels [57,58]. Finally, the TyG revealed one of the lowest accuracies in our survey (AUC = 0.674), in contrast to other investigations related to predicting MASLD onset in the general population (AUC = 0.723) [59]. Furthermore, TyG-related indices have shown significant predictive values for the risk of incident cardiovascular disease and mortality in individuals with MASLD [60]. Regarding cholesterol ratios, TG/HDL showed an AUC = 0.701, while LDL/HDL showed an AUC = 0.656. In a recent analysis, the TG/HDL ratio showed a high AUC (0.747) for detecting MASLD. However, its performance was not significantly different from TG or HDL alone [61]. In addition, Colantoni et al. found this ratio to have a good accuracy (AUC = 0.721) in predicting MASLD onset in the general population [58]. LDL/HDL is also associated with NAFLD onset in people with a normal lipid profile [62]. Finally, the FIB-4 index exhibited the lowest diagnostic accuracy in our analysis, with an AUC = 0.562. In this context, Ferraz-Amaro et al. reported elevated FIB-4 scores in IBD patients presenting with metabolic syndrome; however, no significant correlation was found with disease phenotype, clinical activity scores, or liver stiffness measurements. These findings suggest that the FIB-4 may have limited utility in evaluating hepatic fibrosis in the IBD population, emphasizing the necessity for more accurate and disease-specific fibrosis biomarkers [63].

### 4.3. Limitations

The findings of this study shed light on key metabolic factors contributing to MASLD in individuals with IBD, enhancing our understanding of the disease’s underlying pathophysiological processes within this specific patient group. However, several limitations should be acknowledged. First is its cross-sectional design, which does not allow us to elucidate the temporal relationship between cardiometabolic alterations and liver disease progression [64]. Second, although we employed widely accepted non-invasive indices, transient elastography was not performed. Third is the absence of a MASLD-only group and the evaluation of potential confounders, such as physical activity, dietary habits, and genetic predisposition. Future studies should integrate these variables so as to provide a more comprehensive assessment of their application in the clinical setting.

## 5. Conclusions

Our findings suggest that MASLD in IBD is primarily driven by cardiometabolic dysfunction, with obesity, insulin resistance, and visceral adiposity playing central roles. Given the increased severity of liver disease and the higher prevalence of cardiometabolic features in IBD-MASLD patients, early screening and targeted interventions are warranted in order to prevent liver-related and cardiovascular risks. Additional prospective investigations are essential to the clarification of the underlying mechanisms connecting IBD and MASLD and to the design of targeted therapeutic approaches tailored to this high-risk population. Finally, our findings support the clinical utility of well-known parameters such as BMI and waist circumference, while also identifying other scores such as the METS-IR, LAP, and TG/HDL as potential predictors of MASLD onset in patients with IBD. Therefore, their implementation into routine clinical assessments could prove useful in preventing liver and cardiovascular complications in this setting.

## Figures and Tables

**Table 1:** Comparison of the clinical features of the enrolled patients.

	IBD-NAFLD (*n* = 14)	IBD-MASLD (*n* = 67)	IBD (*n* = 277)	*p*-Value
**Demographic and anthropometric**				
Age (years)	37 ± 15	52 ± 12	46 ± 16	**<0.001**
Male sex, *n* (%)	13 (93)	48 (72)	151 (54)	**<0.001**
Active smokers, *n* (%)	0	4 (6)	33 (12)	0.154
BMI (kg/m^2^)	22 ± 2	27 ± 4	24 ± 4	**<0.001**
Waist circumference (cm)	85 ± 4	100 ± 11	90 ± 11	**<0.001**
LAP	18 ± 5	50 ± 29	31 ± 22	**<0.001**
VAI	1 ± 0.2	2 ± 1	1 ± 1	**<0.001**
**Disease characteristics**				
Disease duration (years)	16 ± 11	14 ± 11	12 ± 11	0.369
Age at onset (years)	22 ± 10	38 ± 15	34 ± 15	**<0.001**
Crohn’s disease, *n* (%)	6 (43)	22 (33)	97 (35)	0.772
CD (Harvey–Bradshaw index)	3 ± 2	5 ± 2	6 ± 3	0.092
Ulcerative colitis, *n* (%)	8 (57)	45 (67)	180 (65)	0.772
UC (full Mayo Score)	2 ± 1	2 ± 1	3 ± 2	0.109
Active disease, *n* (%)	2 (14)	18 (27)	84 (30)	0.396
Extraintestinal manifestations, *n* (%)	3 (21)	13 (19)	34 (12)	0.228
Mild steatosis, *n* (%)	7 (50)	37 (55)	-	**<0.001**
Moderate steatosis, *n* (%)	6 (42)	23 (34)	-	
Severe steatosis, *n* (%)	1 (7)	7 (10)	-	
Surgery, *n* (%)	2 (14)	15 (22)	44 (16)	0.492
**CD disease location and phenotype, *n* (%)**				
Ileal *	4 (67)	10 (45)	57 (59)	0.736
Colonic *	2 (33)	4 (18)	8 (9)	
Ileo-colonic *	0	8 (36)	31 (32)	
Upper GI *	0	0	1 (1)	
Inflammatory *	5 (83)	7 (32)	41 (42)	0.075
Fistulizing *	1 (17)	5 (23)	32 (33)	
Stenosing *	0	10 (45)	24 (25)	
**UC disease location, *n* (%)**				
Proctitis *	1 (12)	3 (7)	18 (10)	0.992
Proctosigmoiditis *	2 (25)	12 (27)	46 (25)	
Left side *	1 (12)	9 (20)	31 (17)	
Pancolitis *	4 (50)	21 (47)	85 (47)	
**Cardiometabolic comorbidities, *n* (%)**				
T2DM	0	9 (13)	9 (3)	**0.002**
Hypertension	0	24 (36)	36 (13)	**<0.001**
Dyslipidemia	0	10 (15)	23 (8)	0.11

Abbreviations: IBD, inflammatory bowel disease; NAFLD, non-alcoholic fatty liver disease; MASLD, metabolic dysfunction-associated steatotic liver disease; BMI, body mass index; LAP, lipid accumulation product; VAI, visceral adiposity index; CD, Crohn’s disease; UC, ulcerative colitis; T2DM, type 2 diabetes mellitus. * The *p*-value was evaluated with regard to CD and UC patients, respectively.

**Table 2:** Comparison of the laboratory parameters, scores, and medications of the enrolled patients.

	IBD-NAFLD (*n* = 14)	IBD-MASLD (*n* = 67)	IBD (*n* = 277)	*p*-Value
**Laboratory parameters and scores (mean ± SD)**				
ALT (UI/L)	24 ± 9	27 ± 26	19 ± 11	**0.001**
AST (UI/L)	25 ± 9	23 ± 12	20 ± 13	**0.017**
Total cholesterol (mg/dL)	175 ± 20	173 ± 43	169 ± 43	0.320
LDL (mg/dL)	108 ± 23	110 ± 37	103 ± 37	0.106
HDL (mg/dL)	58 ± 12	49 ± 13	57 ± 15	**0.021**
TG (mg/dL)	82 ± 15	118 ± 52	95 ± 46	**<0.001**
Fasting blood glucose (mg/dL)	87 ± 7	93 ± 21	86 ± 14	**<0.001**
Fasting insulinemia (mg/dL)	7 ± 1	11 ± 8	8 ± 5	**<0.001**
CRP (mg/L)	5 ± 3	7 ± 12	8 ± 12	0.442
Platelets (×10^3^/uL)	289 ± 185	267 ± 123	250 ± 102	0.772
Fecal calprotectin (mcg/gr)	314 ± 457	403 ± 833	696 ± 1427	0.191
HOMA-IR	1.5 ± 0.4	3 ± 2	2 ± 1.5	**<0.001**
METS-IR	31 ± 4	40 ± 8	33 ± 6	**<0.001**
TyG	8 ± 0.2	8 ± 0.4	8 ± 0.5	**<0.001**
TG/HDL	1.5 ± 0.5	3 ± 2	2 ± 1	**<0.001**
LDL/HDL	2 ± 1	3 ± 1	2 ± 1	**<0.001**
FIB-4	1 ± 0.5	1 ± 0.5	1 ± 1	0.546
**Medications**				
Salicylates, *n* (%)	6 (43)	37 (55)	148 (53)	0.700
Azathioprine, *n* (%)	1 (7)	4 (6)	37 (13)	0.208
>3 cycles of steroids, *n* (%)	1 (7)	6 (9)	22 (8)	0.955
Biological therapy, *n* (%)	7 (50)	28 (42)	101 (36)	0.529
Anti-TNF-α, *n* (%)	6 (86)	20 (71)	65 (64)	0.174
Vedolizumab, *n* (%)	1 (14)	3 (11)	26 (26)	0.428
Ustekinumab, *n* (%)	0	5 (18)	10 (10)	0.268
>1 Biological drug, *n* (%)	0	6 (9)	20 (7)	0.501
Current biological therapy duration (years)	5 ± 3	3 ± 2	2 ± 2	0.209
Total biological therapy duration (years)	7 ± 2	4 ± 3	4 ± 4	0.507

Abbreviations: IBD, inflammatory bowel disease; NAFLD, non-alcoholic fatty liver disease; MASLD, metabolic dysfunction-associated steatotic liver disease; ALT, alanine aminotransferase; AST, aspartate aminotransferase; LDL, low-density lipoprotein; HDL, high-density lipoprotein; TG, triglycerides; CRP, C-reactive protein; HOMA-IR, homeostasis model assessment of insulin resistance; METS-IR, metabolic score for insulin resistance; TyG, triglyceride-glucose index; TG/HDL, triglyceride/high-density lipoprotein; LDL/HDL, low-density lipoprotein/high-density lipoprotein; FIB-4, fibrosis-4; TNF-α, tumor necrosis factor-alfa.

**Table 3:** Dunn’s post hoc analysis performed among the groups.

	IBD-NAFLD (*n* = 14)	IBD-MASLD (*n* = 67)	IBD (*n* = 277)	IBD-NAFLD vs. IBD-MASLD *p* Value	IBD-NAFLD vs. IBD *p* Value	IBD-MASLD vs. IBD *p* Value
**Demographic and anthropometric**						
Age (years)	37 ± 15	52 ± 12	46 ± 16	**<0.001**	0.089	**<0.001**
BMI (kg/m^2^)	22 ± 2	27 ± 4	24 ± 4	**<0.001**	0.096	**<0.001**
Waist circumference (cm)	85 ± 4	100 ± 11	90 ± 11	**<0.001**	0.110	**<0.001**
LAP	18 ± 5	50 ± 29	31 ± 22	**<0.001**	0.179	**<0.001**
VAI	0.9 ± 0.2	2 ± 1	1 ± 1	**0.005**	0.286	**<0.001**
**Disease characteristics**						
Disease duration (years)	16 ± 11	14 ± 11	12 ± 11	0.875	0.431	0.214
Age at onset (years)	22 ± 10	38 ± 15	34 ± 15	**<0.001**	**0.025**	**0.001**
CD (Harvey–Bradshaw index)	3 ± 2	5 ± 2	6 ± 3	0.175	**0.041**	0.323
UC (full Mayo Score)	2 ± 1	2 ± 1	3 ± 2	0.315	0.088	0.165
**Laboratory parameters and scores**						
ALT (UI/L)	24 ± 9	27 ± 26	19 ± 11	0.963	0.088	**<0.001**
AST (UI/L)	25 ± 9	23 ± 12	20 ± 13	0.667	0.093	**0.014**
Total cholesterol (mg/dL)	175 ± 20	173 ± 43	169 ± 43	0.992	0.481	0.163
LDL (mg/dL)	108 ± 23	110 ± 37	103 ± 37	0.741	0.502	0.039
HDL (mg/dL)	58 ± 12	49 ± 13	57 ± 15	0.171	0.922	**0.006**
TG (mg/dL)	82 ± 15	118 ± 52	95 ± 46	**0.048**	0.988	**<0.001**
Fasting blood glucose (mg/dL)	87 ± 7	93 ± 21	86 ± 14	0.248	0.444	**<0.001**
Fasting insulinemia (mg/dL)	7 ± 1	11 ± 8	8 ± 5	0.068	0.557	**<0.001**
CRP (mg/L)	5 ± 3	7 ± 12	8 ± 12	0.886	0.768	0.908
Platelets (×10^3^/uL)	289 ± 185	267 ± 123	250 ± 102	0.943	0.790	0.489
Fecal calprotectin (mcg/gr)	314 ± 457	403 ± 833	696 ± 1427	0.920	0.354	0.099
HOMA-IR	1.5 ± 0.4	3 ± 2	2 ± 1.5	0.139	0.441	**<0.001**
METS-IR	31 ± 4	40 ± 8	33 ± 6	**<0.001**	0.409	**<0.001**
TyG	8 ± 0.2	8 ± 0.4	8 ± 0.5	0.063	0.928	**<0.001**
TG/HDL	1.5 ± 0.5	3 ± 2	2 ± 1	**0.028**	0.909	**<0.001**
LDL/HDL	2 ± 1	3 ± 1	2 ± 1	0.265	0.105	**<0.001**
FIB-4	1 ± 0.5	1 ± 0.5	1 ± 1	0.548	0.704	0.769
**Medications**						
Current biological therapy duration (years)	5 ± 3	3 ± 2	2 ± 2	0.733	0.532	0.086
Total biological therapy duration (years)	7 ± 2	4 ± 3	4 ± 4	0.736	0.762	0.248

Abbreviations: IBD, inflammatory bowel disease; NAFLD, non-alcoholic fatty liver disease; MASLD, metabolic dysfunction-associated steatotic liver disease; BMI, body mass index; LAP, lipid accumulation product; VAI, visceral adiposity index; CD, Crohn’s disease; UC, ulcerative colitis; ALT, alanine aminotransferase; AST, aspartate aminotransferase; LDL, low-density lipoprotein; HDL, high-density lipoprotein; TG, triglycerides; CRP, C-reactive protein; HOMA-IR, homeostasis model assessment of insulin resistance; METS-IR, metabolic score for insulin resistance; TyG, triglyceride-glucose index; TG/HDL, triglyceride/high-density lipoprotein; LDL/HDL, low-density lipoprotein/high-density lipoprotein; FIB-4, fibrosis-4.

**Table 4:** ROC analysis performed to predict MASLD onset in patients with IBD.

Variable	AUC (95% CI)	Sensitivity	Specificity	Cut-Off
METS-IR	0.754 (0.694–0.814)	70.5%	70.4%	36.52
Waist circumference (cm)	0.754 (0.696–0.811)	66.7%	77%	93.55
LAP	0.737 (0.675–0.798)	61.5%	78.6%	29.8
BMI (kg/m^2^)	0.709 (0.646–0.773)	62.2%	68.8%	24.99
TG/HDL	0.701 (0.630–0.773)	69.5%	68.8%	1.91
HOMA-IR	0.680 (0.605–0.755)	61.5%	67.2%	1.65
TyG	0.674 (0.598–0.751)	65.3%	65.5%	8.33
VAI	0.664 (0.590–0.737)	62.8%	67.2%	1.22
LDL/HDL	0.656 (0.581–0.730)	63.2%	67.2%	1.96
FIB-4	0.562 (0.491–0.634)	46.8%	67.2%	10.8

Abbreviations: AUC, area under the curve; CI, confidence interval; METS-IR, metabolic score for insulin resistance; LAP, lipid accumulation product, BMI, body mass index; TG/HDL, triglyceride/high-density lipoprotein; HOMA-IR, homeostasis model assessment of insulin resistance; TyG, triglyceride-glucose index; VAI, visceral adiposity index; LDL/HDL, low-density lipoprotein/high-density lipoprotein; FIB-4, fibrosis-4.

## Data Availability

The original contributions presented in the study are included in the article; further inquiries can be directed to the corresponding author.

## Abbreviations

The following abbreviations are used in this manuscript.

ALT	Alanine aminotransferase
AST	Aspartate aminotransferase
AUC	Area under the curve
BMI	Body mass index
CD	Crohn’s disease
CI	Confidence interval
CRP	C-reactive protein
FIB-4	Fibrosis-4 index
HOMA-IR	Homeostasis model assessment of insulin resistance
HDL	High-density lipoprotein
IBD	Inflammatory bowel disease
LAP	Lipid accumulation product
LDL	Low-density lipoprotein
MAFLD	Metabolic dysfunction-associated fatty liver disease
MASLD	Metabolic dysfunction-associated steatotic liver disease
METS-IR	Metabolic score for insulin resistance
NAFLD	Non-alcoholic fatty liver disease
ROC	Receiver operating characteristic
SD	Standard deviation
T2DM	Type 2 diabetes mellitus
TG	Triglycerides
TG/HDL	Triglyceride-to-high-density lipoprotein cholesterol ratio
TNF-α	Tumor necrosis factor-alpha
TyG	Triglyceride-glucose index
UC	Ulcerative colitis
VAI	Visceral adiposity index
